# Radiation-induced sterility for pupal and adult stages of the malaria mosquito *Anopheles arabiensis*

**DOI:** 10.1186/1475-2875-5-41

**Published:** 2006-05-15

**Authors:** Michelle EH Helinski, Andrew G Parker, Bart GJ Knols

**Affiliations:** 1 Entomology Unit, FAO/IAEA Agriculture and Biotechnology Laboratory, International Atomic Energy Agency (IAEA), A-2444 Seibersdorf, Austria; 2 Laboratory of Entomology, Wageningen University and Research Center, P.O. Box 8031, 6700 EH Wageningen, The Netherlands

## Abstract

**Background:**

In the context of the Sterile Insect Technique (SIT), radiation-induced sterility in the malaria mosquito *Anopheles arabiensis *Patton (Diptera: Culicidae) was studied. Male mosquitoes were exposed to gamma rays in the pupal or adult stage and dose-sterility curves were determined.

**Methods:**

Pupae were irradiated shortly before emergence (at 22–26 hrs of age), and adults <24 hrs post emergence. Doses tested ranged between 0 and 100 Gy. The effects of irradiation on adult emergence, male survival, induced sterility and insemination capability were evaluated. Emergence and insemination data were analysed using independent t-tests against the control. Correlation analyses were performed for insemination rate and dose and insemination and fecundity. Male survival was analysed using Kaplan-Meier survival analyses. Finally, the calculated residual fertility values were inverse-normal transformed and linear regression analyses performed.

**Results:**

Irradiation of pupae, for all doses tested, had no effect on adult emergence. Survival curves of males irradiated as pupae or adults were similar or even slightly higher than non-irradiated males. Overall, adults appeared to be slightly more susceptible to irradiation, although no significant differences for individual doses were observed. In the pupal stage, a significant negative correlation was found between insemination and dose, but the correlation-coefficient was associated with less than 25% of the total variation. A review of the literature indicated that *An. arabiensis *is more radiation resistant than other anopheline mosquitoes.

**Conclusion:**

The optimal dose for male insects to be released in an SIT programme depends on their level of sterility and competitiveness. The use of semi-sterilizing doses to produce more competitive insects is discussed. The most convenient developmental stage for mosquito irradiation on a mass-scale are pupae, but pupal irradiation resulted in a lower insemination rate at the highest dose compared to adult irradiation. On the basis of this study, a suitable dose range that includes semi-sterilizing doses is identified to initiate competitiveness experiments for males irradiated at both developmental stages.

## Background

In the 21^st ^century, anopheline mosquitoes remain the most deadly insects in the world. Malaria is still widely spread; it is estimated that currently 3.2 billion people live in areas at risk of malaria transmission [1]. Estimated economic growth reduction in endemically affected countries is high, and contemporary control methods are not always effective due in part to widespread resistance of the mosquitoes to insecticides and *Plasmodium *parasites to commonly used drugs. The Sterile Insect Technique (SIT), successfully applied against a number of pest species [2], has been evaluated against *Anopheles albimanus *Wiedemann in the 1970s with encouraging results [3,4]. Over the last years, a renewed interest in SIT for malaria vectors has led to a 5-year feasibility study to investigate all aspects of an SIT programme including sexing, mass production, sterilisation, and release methodology [5,6]. The project initially focuses on the African malaria vector *Anopheles arabiensis *Patton.

The SIT relies on the sterilisation of insects by chemosterilisation [4,7,8], irradiation [2] or modern biotechnological approaches [9-11]. Modern biotechnological approaches based on transgenic organisms are promising but at an early stage of development and no legal framework yet exists to facilitate the introduction of such organisms in the wild [12,13]. Sterilisation by irradiation or chemosterilants has not been researched extensively for the last 30 years with mosquitoes. Promising results were obtained with chemosterilants in terms of the level of sterility induced and competitiveness present [8] but these have the disadvantage of being mutagenic agents. They thus present a potential hazard to humans during the treatment process and non-target organisms if residues persist in released individuals [8]. Even though the actual amount of residue released in the environment was in fact very low due to careful rinsing of the pupae [14], concerns raised about the possible negative effects on the environment if large numbers of treated insects were to be released [15,16] resulted in a disuse of chemosterilants for mosquito control. Although it would be worthwhile to identify additional compounds with chemosterilant properties, it remains doubtful if the currently available ones will be acceptable for use in future genetic control programmes.

Sterilisation by irradiation remains the most practical way to sterilise the insects at present, and it has been argued that radiation sterilisation should also be used to introduce the first transgenic organisms in the wild [3]. Determining the optimal dose range for an SIT programme depends on the level of sterility induced and the competitiveness of the irradiated males. A low dose may result in insufficiently sterilised males, whereas a high dose may undermine the insect's ability to compete with wild con-specifics and may thus reduce the overall impact of the release.

In the context of SIT, anopheline irradiation has been performed on a number of species and dose-response curves have been published for *An. albimanus *[17], *An. pharoensis *Theobald [18,19] and *An. stephensi *Liston [7,20]. *An. arabiensis *has been studied in the light of genetic sexing systems [21] and small-scale irradiation studies [22] but no dose-response curve exists. Previous work has indicated that substantial inter-species variation in radiation sensitivity is present [22], justifying the need for a dose-sterility curve for *An. arabiensis*. In mosquitoes, both the pupal and the adult stage can be irradiated. Pupal irradiation is easier to perform, but there is evidence of a reduced competitiveness when male pupae are irradiated at high doses compared to adult irradiation [23]. The objective of this study was to determine the dose-sterility curves for the pupal and adult stages of male *An. arabiensis *and define a suitable dose range to initiate competitiveness experiments.

## Methods

### Mosquitoes

The mosquito strain used is the "KGB" strain of *An. arabiensis*. The strain originates from Zimbabwe and has been colonized since 1975 (Courtesy of MR4, CDC Atlanta, USA). All mosquitoes used in the experiments were reared at a density of ~750 larvae per tray (30 × 40 cm) containing ± 2 liter of deionized water (water depth 2 cm). Heating mats were used to maintain the water temperature at 28°C. Larvae were fed a diet of fish-food (Aquaricare^®^) daily (~0.3 mg/larva) that was powdered and passed through a 224 μm mesh sieve. Adults were maintained in the insectary at 28°C and 80% RH and all experiments were conducted in standard 30 cm cubic cages. The light regime was L10:D12 with a one hour simulated dusk and dawn period. Adult cages were continuously supplied with 10% sucrose solution [w/v].

### Irradiation procedure

**Figure 1:**
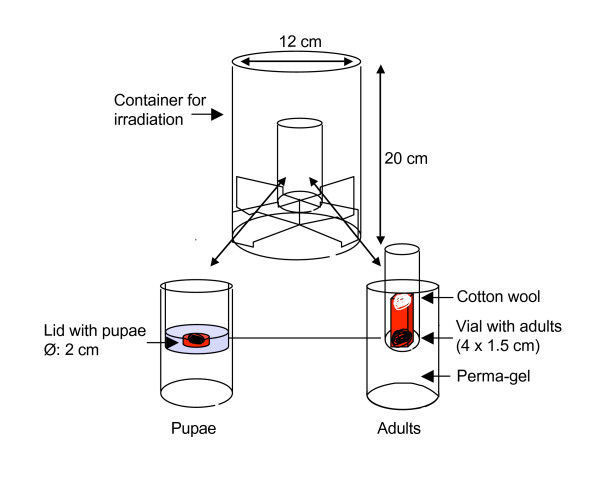
Insects were irradiated in a confined space in the centre of the irradiation chamber to minimize maximise dose uniformity within the batch. Devices used for the irradiation of pupae and adults are shown.

Insects were exposed to gamma rays generated by a cobalt-60 source (Gammacell 220, MDS Nordion, Ottawa, Canada) with a dose rate of ca 16 Gy/min. Insects were concentrated in the centre of the chamber to maximise dose uniformity within the batch (Fig. 1). A dosimetry system was used to measure the dose received by the batch based on the Gafchromic HD-810 film (International Specialty Products, NJ, USA) [24]. Three dosimeters were included with each batch of insects and read after irradiation with a Radiachromic^® ^reader (Far West Technology, Inc., California, USA).

### Experimental set-up

Experiments were performed in series. Each series included a number of different irradiation doses and a control (Table 1). The experimental mosquitoes in each series originated from the same batch of material and were randomly distributed to treatment. Mosquitoes in the control group underwent exactly the same handling stages as the experimental mosquitoes, except the actual irradiation itself. Within each series, order of irradiation was assigned randomly. We covered a spectrum of doses that ranged from a control (no irradiation) to a dose previously shown to yield almost complete sterility [7,25]. These were 0, 25, 50, 60, 70, 80, and 100 Gy for the pupal and adult experiments. In the pupal experiments, two additional doses of 35 and 45 Gy were included. Each dose was replicated twice, and the doses between 50 and 80 were replicated three times.

**Table 1:** Effect of different irradiation doses on median (± SE) survival times of *An. arabiensis *males. Log-rank tests compared treatments with the control. Survival time was estimated from Kaplan-Meier survival analysis (details see text).

Pupae Series	Dose (Gy)	n	Median survival time	Log-Rank	Adult Series	Dose (Gy)	n	Median survival time	Log-Rank
1	0	31	13 ± 0.6	Na	1	0	57	7 ± 0.8	Na
	25	35	7 ± 1.7	14.66**		60	55	10 ± 0.8	1.59
	35	27	11 ± 1.0	6.09*		70	56	10 ± 0.6	5.10*
2	0	56	9 ± 1.1	Na		80	52	10 ± 0.9	0.04
	50	48	10 ± 0.2	0.84	2	0	54	8 ± 0.8	Na
	70	55	6 ± 2.9	0.10		50	59	10 ± 1.1	0.07
3	0	48	11 ± 1.5	Na		60	51	10 ± 0.9	0.16
	80	51	13 ± 2.3	4.93*		70	56	7 ± 1.2	0.11
	100	61	11 ± 1.7	1.63		80	50	10 ± 1.3	2.60
4	0	46	6 ± 1.5	Na		100	55	10 ± 0.9	0.00
	35	54	13 ± 0.8	2.70	3	0	49	10 ± 1.9	Na
	45	58	11 ± 0.5	1.08		25	48	10 ± 1.7	0.84
	50	53	11 ± 1.1	0.58		50	45	10 ± 1.1	0.05
5	0	32	10 ± 2.2	Na		100	47	10 ± 1.7	0.44
	60	45	10 ± 0.8	0.34	4	0	48	6 ± 2.7	Na
	70	35	10 ± 2.1	1.29		25	48	15 ± 2.4	1.06
	80	35	12 ± 0.9	0.77		50	48	12 ± 2.2	1.79
6	0	44	7 ± 1.2	Na		60	48	11 ± 1.5	0.29
	45	42	10 ± 2.2	3.74		70	46	11 ± 1.2	0.59
	60	42	4 ± 1.3	0.19		80	49	11 ± 4.2	0.00
	70	41	10 ± 1.6	2.32					
	80	41	10 ± 1.0	2.94					
	100	43	10 ± 1.2	0.70					
7	0	44	10 ± 1.4	Na					
	25	45	12 ± 1.7	10.63**					
	45	42	12 ± 0.6	11.57**					
	50	41	10 ± 0.3	0.07					
	60	45	11 ± 0.7	5.73*					
	50	42	12 ± 0.9	8.10**					

Na: not applicable; *: P < 0.05; **: P < 0.01

### Parameters measured

#### Emergence

Emergence from irradiated pupae was scored for both sexes, as pupae were not sexed prior to irradiation. Only mosquitoes that successfully emerged were positively scored; semi-emerged adults and dead adults were scored as non-emerged.

#### Longevity

The longevity of the irradiated mosquitoes was determined by the removal and counting of dead mosquitoes at 24 hr intervals (except during weekends, when 48–72 hr intervals were used). Cages were discarded when at least two-third of all the males had died.

#### Sterility assessment

Levels of sterility were observed by mating the irradiated males with non-irradiated virgin females. To ensure virginity of females, pupae were placed in individual tubes prior to emergence. Mates were introduced into experimental cages in a 1:1 ratio. For experiments with pupae, mates were introduced into the cages the day after irradiation when the males had emerged. For the adult experiments, mates were introduced after the irradiation on the same day. Mosquitoes were fed on the forearm of a volunteer for 10 min twice on consecutive days, between day 2 and 5 after introduction of the females (with the exception of series 1 in the adult stage when mosquitoes were fed on a membrane filled with human blood). Egg laying occurred *en masse *in the cage. For each cage, one egg bowl filled with water and lined with wet filter paper was offered for five nights starting two days after the first blood meal. Daily, or at 48 hr intervals, eggs were removed and counted. For hatch rate determination, the eggs were thoroughly mixed, and random samples of eggs were placed in larger trays to allow hatching. Trays from control cages were filled with ca. 200 eggs/tray, and more eggs were placed in one tray as the doses increased due to higher sterility levels of the eggs. Eggs were checked for their hatchability by counting and removing L1 larvae daily for seven consecutive days from the trays.

For each day of egg collection, all eggs or a sample of all eggs were used to determine hatch rates. Since eggs were thoroughly mixed, the sample can be regarded as representative of the total amount laid for that day and treatment, therefore hatch rates were weighted to the total number of eggs collected for that day. For each treatment, an average value was obtained per series by weighting the data for all egg collection days. The residual fertility was calculated as a percentage of the control fertility of each series and subtracted from 100% (Abbott's formula [26]) to give a value for radiation-induced sterility.

Fecundity (the average number of eggs laid per female) was calculated by dividing the number of eggs laid per night by the number of females alive at the start of that night. A value for each treatment per series was obtained by the sum of all egg laying nights and data were pooled per treatment for both stages.

#### Insemination

The proportion of females inseminated by irradiated males was extrapolated from dissections of a sub-sample of females tested. After egg laying, a random selection of females was taken from the cages and their spermatheca dissected to examine whether these had been inseminated. The presence of spermatozoa was confirmed using a compound microscope at 400× magnification.

### Collection and irradiation of experimental mosquitoes

#### Pupae

Pupae were collected the day before irradiation at 3 pm from trays that had been cleared of all their pupae before 9 am that day to ensure equal age of the pupae. At 11 am the next day, the pupae, aged 22–26 hrs, were irradiated. Pupae were irradiated in a small plastic lid filled with water (Fig. 1). For each dose, 100 pupae were irradiated at once. After irradiation, individual pupae were put in small vials and left overnight for emergence. The following morning, males were transferred to the cages according to treatment.

#### Adults

Irradiation of adults was performed on specimens < 24 hrs of age. Pupae were collected from trays and allowed to emerge overnight in a standard cage. The following morning, the mosquitoes were separated by sex and males were placed in a small holding cup prior to irradiation and sugar water was offered on cotton wool. Fifty adult males were irradiated for each dose. Adults were irradiated in a modified version of the pupal irradiation device (Fig. 1). Perma-gel^® ^from an ice-pack (Ice-pak™, Cryopak Industries Inc., Canada) surrounded a tube in which a small vial was placed that contained the adults. The irradiation device was placed at ~4°C prior to irradiation to cool the gel and immobilize the adults during the irradiation. Adults were immobilized in an ice-box (~4°C) for 5 min before irradiation and transferred to the vial. The opening of the vial was closed with some cotton wool and placed in the irradiation device. After irradiation, the vial was opened inside the cage and the adults were left to recover and disperse.

### Statistical analysis

Data was analysed using the following variables: treatment (dose: 0, 25, 35, 45, 50, 60, 70, 80, 100 Gy), series (1–7), and stage (pupae or adults). Unless stated otherwise, for both stages, data from the same treatments between different series were pooled to get an average value per treatment. Proportional values for adult emergence and insemination were arcsine-square-root-transformed to achieve normal distribution. Independent t-tests were used to determine significant differences between the mean values of treated groups and the control. In addition, correlation analyses (Pearson correlation coefficient) between dose and insemination rate, and between fecundity and insemination rate were performed for both stages.

Survival curves were analysed using Kaplan-Meier survival analyses. The obtained survival curves were compared to the control using Mantel-Cox log-rank tests.

General Linear Models (GLM) were used to observe differences in fecundity between treatments for the two stages, and to compare induced sterility levels between stages.

The calculated residual fertility values were inverse-normal transformed to yield normal equivalent deviates (N.E.D.) and irradiation doses were log_10 _transformed to obtain a linear relationship between dose and residual fertility. A standard linear regression analysis was performed for each stage with log_10 _(Dose/Gy) as the independent and N.E.D. as the dependent variable.

All two-sided tests were performed using SPSS version 12 (SPSS Inc., Chicago, IL).

## Results

Dosimetry confirmed that all doses delivered lay within a 5% error range.

### Emergence

**Figure 2:**
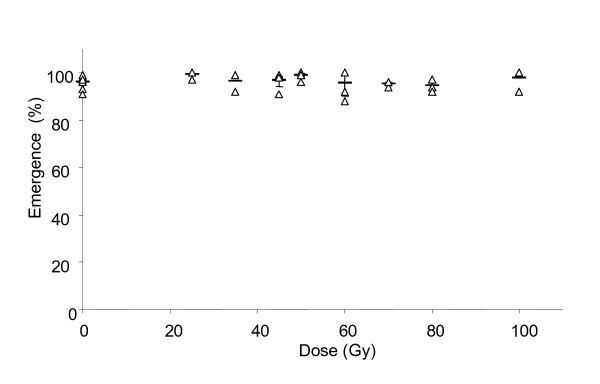
Adult emergence for the different treatments. Triangles indicate individual values; detransformed means (± SE, if n>2) are indicated by a horizontal line.

Irradiation of 20–26 hr old pupae had no effect on adult emergence. Overall, emergence was high (Fig. 2), on average 96 ± 0.6%. No significant differences were observed between adult emergence for the different treatments compared to the control (independent t-tests, data not shown).

### Induced sterility

The total number of eggs laid in all experiments was ~78600 of which ~46600 eggs were checked to estimate the proportion that had hatched. The average number of eggs laid per female in all treatments was similar to the control for both irradiated life stages (pupae: (F_8,20 _= 0.65, p > 0.05); adults: (F_6,13 _= 0.74, p > 0.05), (Table 2).

**Table 2:** Mean (± SE) induced sterility levels and mean (± SE) fecundity of females mated with *An. arabiensis *males irradiated in the pupal or adult stage. A range is indicated where number of replicates is <2 (n: total number of females alive at onset of egg-laying period; Nd: not done).

Treatment Dose (Gy)	Pupal irradiation				Adult irradiation			
	
	Replicates	Induced sterility	Fecundity	n	Replicates	Induced sterility	Fecundity	n
0	7	0.0	45 ± 4.9	266	4	0.0	42 ± 11.6	169
25	2	35.4 (32.3–38.5)	32 (25–38)	56	2	38.9 (38.7–39.0)	69 (62–76)	84
35	2	44.8 (35.7–53.8)	52 (49–54)	59	Nd			
45	3	68.3 ± 6.9	42 ± 5.7	125	Nd			
50	4	76.0 ± 4.6	34 ± 5.0	178	3	71.7 ± 5.8	46 ± 11.0	135
60	3	78.6 ± 5.7	39 ± 8.6	104	3	88.2 ± 2.2	29 ± 13.4	134
70	3	83.4 ± 2.1	41± 12.0	120	3	92.4 ± 2.5	40 ± 16.2	130
80	3	91.0 ± 3.4	42 ± 5.5	104	3	96.7 ± 0.5	34 ± 10.7	115
100	2	98.6 (98.5–98.7)	34 (26–41)	101	2	98.1 (97.2–98.9)	41 (21–60)	86

**Figure 3:**
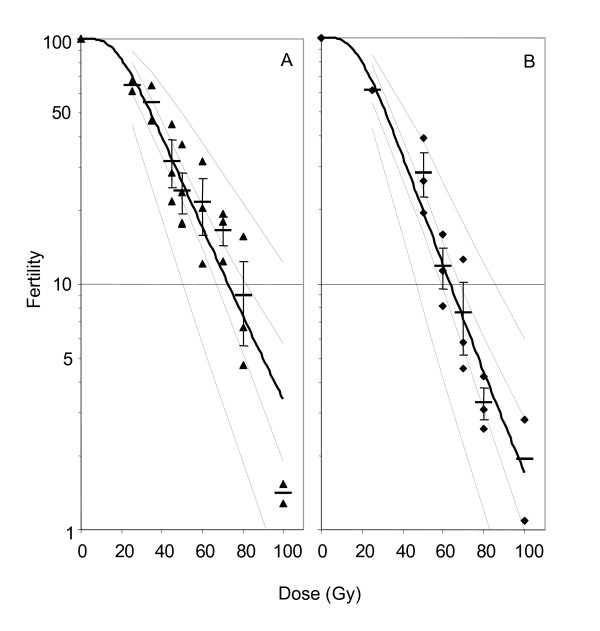
Regression line (± 95% CI for mean and individual values) for fertility versus irradiation dose (Gy) for male *An. arabiensis *pupae (A) and adults (B). Symbols indicate observed individual values. Means (± SE, if n>2) are indicated by a horizontal line.

Control sterility (i.e. the number of eggs that naturally do not hatch) in the KGB colony is 26 ± 9%. Corrected for this control sterility, the residual fertility decreased with increasing radiation dose for both pupal and adult stages (Fig. 3). A linear regression (pupae (F_1,20 _= 105.3, p < 0.01; adults (F_1,14 _= 142.3, p < 0.01)) was obtained after transformation of both axes, and the regression model can be used to predict induced sterility rates (= 1 - residual fertility) at specific irradiation doses.

For the pupal stage the equation is:

N.E.D. (residual fertility) = 6.07 - 3.95(log_10_(dose/Gy)) with r^2 ^= 84%;

for the adults:

N.E.D. (residual fertility) = 6.33 - 4.22(log_10_(dose/Gy)) with r^2 ^= 91%.

Plotting fertility on a logarithmic scale against dose provides insight in the nature of dominant lethal mutations. A linear response indicates a "one-hit" relationship whereas departures from linearity indicate a "multi-hit" relationship [27,28]. Graphs for the pupal and adult stage show a predominantly linear relationship (Fig. 3) suggesting a one-hit relationship to dose; i.e. a large proportion of dominant lethal mutations result from single events in the gametes. As expected, at higher doses the lines tend to depart from linearity suggesting that gametes carry more than one dominant lethal mutation.

**Figure 4:**
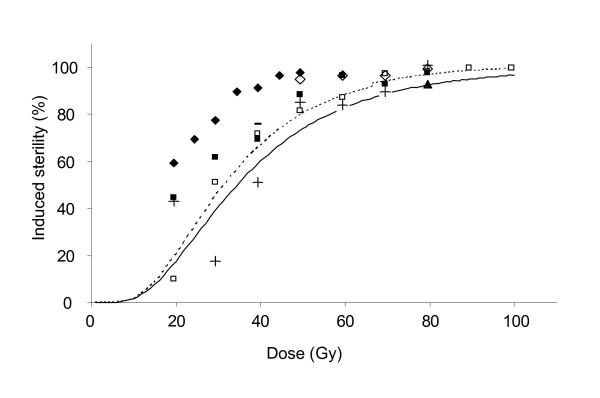
Comparative analysis of dose-sterility data from this study (solid line for *An. arabiensis *pupae, dotted line for adults) and published reports on anopheline irradiation (using 20–100 Gy). Induced sterility levels were calculated from observed sterility levels for [7,18]. +:*An. albimanus *pupa [17]; □: *An. stephensi *pupa [7]; ■: *An. stephensi *adult [20]; ◇: *An. pharoensis *pupa [18]; ◆: *An. pharoensis *adult [19]; ▲: *An. gambiae *adult [22]; -*An. arabiensis *adult [21].

When comparing the two stages, germ cells of adults were more susceptible to irradiation resulting in higher induced sterility levels (GLM: Stage F_1,24 _= 4.55, p < 0.05) (Fig. 4). More specifically, irradiation of adults yielded higher sterility than irradiation of pupae at doses between 60 and 80 Gy (Table 2) although no significant differences were observed between the two stages for each dose individually (independent t-tests, data not shown).

### Mosquito survival

The recovery of adults after irradiation was 100%. In total, survival was scored for 2303 males. Because the series controls were significantly different in the pupal (Log-rank = 18.76, df = 6, p ≤ 0.01), and adult stage (Log-rank = 13.1, df = 3, p ≤ 0.01), survival curves were analysed per series against the control. For the pupal stage, the survival curve for irradiated males was not significantly lower than the control, except in series 1 where males irradiated with 25 and 35 Gy had lower survival (Table 1). Higher survival was also found in certain treatments. In the adult stage, survival of irradiated males was similar to the control, with the exception of 70 Gy males in series 1, for which survival was higher (Table 1).

### Insemination

**Figure 5:**
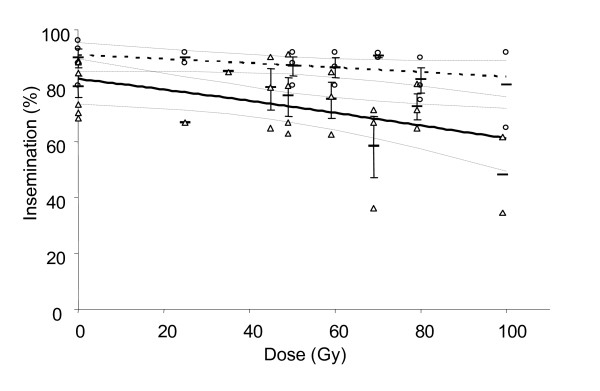
Insemination of virgin females mated with *An. arabiensis *males irradiated in pupal or adult stage for the different treatments. Triangles represent pupal individual values, circles adult individual values. Detransformed means (± SE if n>2) are indicated by a horizontal line. Linear regression lines (± 95% CI) are given; solid line is pupae, dotted line adults. For clarity purposes, data points for pupae have been shifted slightly to the left.

A total number of 999 females was dissected (on average 21.7 ± 0.6 females per cage) and examined for insemination (except in series 1 for the pupal stage; no dissections were performed). After on average ten days of mating, control insemination was 79% ± 4 for females confined with males irradiated at the pupal stage, and 89% ± 4 in for those with males irradiated in the adult stage, and no significant differences were observed between both controls (t_s _= 0.40, df = 8, p = > 0.05). For the pupae, a significant negative correlation (r = -0.47, p < 0.01) was found between dose and insemination (Fig. 5). For adults, no significant correlation was observed (r = -0.37, p > 0.05). Individual t-tests showed that insemination rates were not statistically different from the control for all treatments in both stages, except at 100 Gy for the pupal stage (t_s _= 3.11, df = 6, p < 0.05). However, when comparing the two stages at individual doses, no significant differences were found (independent t-tests, data not shown). A positive correlation was found between fecundity (number of eggs/female) and insemination of females placed together with males irradiated in the pupal stage (r = 0.67, p < 0.01), (r^2 ^= 45%).

## Discussion

Ionizing radiation has, over the years, proven to be a safe and reliable way to induce sterility in a large variety of insects [2]. The potential use of the SIT against malaria vectors is currently explored and the development of radiation sterilisation protocols is a vital part of such endeavour.

Most experimental work on *Anopheles *irradiation has focused on the pupal stage [7,17,18,25,29]. The irradiation of pupae is preferably performed on older (>15 hrs) pupae since irradiation of young pupae results in a reduced emergence [22,25] and shorter survival [30]. In this study, age of the pupae when irradiated was between 22–26 hrs and ~7–12 hrs before emergence. We found that the irradiation had no effect on the emergence from the pupae even when the dose applied was high. Similar results were reported for pupal irradiation of *An. pharoensis *[25].

Although irradiation is intended to target the germ cells, the process is non-specific and somatic cells may also be damaged. One of the commonest effects of somatic damage is a reduced longevity [31]. In this study, males irradiated as pupae or adults with increasing doses had similar or even higher survival times compared to the controls. Only in two cases a reduced survival was found, but as this was observed at the lowest doses, irradiation is not the likely cause. For *An. pharoensis *[32], a slight increase in longevity of males irradiated with 5–70 Gy as pupae compared to the control was reported. Other studies report a reduced longevity; in *An. pharoensis *[25] for pupae irradiated at 100–130 Gy, and in *A. stephensi *[7] for pupae irradiated at 80 and 120 Gy. Sampling of mosquitoes occurred at 48–72 hrs intervals over the weekends, which could have influenced consistency of the data to some extent. However, as the controls were exposed to the same treatment and subsequent analyses we deem this variation caused by different sampling intervals of negligible importance.

The fecundity of the females mated with irradiated males was similar for all treatments compared to the control. Due to lower insemination of females placed with males irradiated at higher doses in the pupal stage, a positive correlation was observed between fecundity and insemination rate. Overall, fecundity rates were variable because eggs were collected *en masse *and fecundity calculated over all females alive regardless if they had blood fed or oviposited. Differences observed in fecundity are partly accounted for by the reduced insemination rate found in certain treatments.

Unlike in some other mosquito colonies, uninseminated females of the KGB strain used in these experiments have not been observed to oviposit, resulting in fewer eggs when the insemination rate is low. Other, inexplicable variation in egg batch sizes have been observed, yet should not influence our conclusions since these occurred throughout the experimental period and across all treatments.

The dose-sterility curves in *An. arabiensis *for pupae and adults show the classic pattern found for such curves of a linear relationship at low doses and the flattening of the curve at higher doses [33,34]. The highest dose of 100 Gy induced > 98% sterility. Overall, we found that fertility is slightly more sensitive to exposure in the adult stage than in the pupal stage. There are only a few studies with anophelines that have irradiated both stages simultaneously. At 120 Gy, equal levels of sterility were found for pupal and adult irradiation in *An. gambiae *and *An. stephensi *[23]; while in *An. gambiae *pupae were more radiation-resistant than adults [22]. If we compare levels of sterility found in different studies, *An. stephensi *[20] and *An. pharoensis *[19] both show higher sterility levels in the adult stage (Fig. 4).

When comparing the level of induced sterility in *An. arabiensis *with other anophelines (Fig. 4), *An. arabiensis *shows a greater radiation resistance resulting in lower induced sterility levels. In the pupal stage, *An. albimanus *[17] behaved similarly at doses > 40 Gy, while *An. stephensi *[7] and *An. pharoensis *[18] overall had slightly higher induced sterility levels. In the adult stage, *An. pharoensis *[19] and especially *An. stephensi *[20] showed higher sterility levels. Only in *An. gambiae *[22] at 80 Gy a lower level of induced sterility was observed. In a study in which adult *An. arabiensis *were irradiated with 40 Gy [21], a somewhat higher level of sterility was reported, but the causes for this remain unclear.

Although competitiveness experiments will be used to assess male fitness at different radiation doses, the level of insemination in the absence of competition gives some indication of the male's ability to mate. We found a substantial variation in insemination within some treatments, but the number of replicates was low. The insemination of females mated with males irradiated as adults was comparable to the control. For males irradiated at the pupal stage, a negative correlation was found between insemination and dose but the correlation-coefficient (r^2^) was associated with less than <25% of the total variation and only the dose of 70 Gy was different to the control. When the two stages were compared at individual doses, no differences were observed. In previous studies, equal insemination [17] or similar egg production [18,25] in females mated to males irradiated as pupae was observed. The results in this study suggest a decline in mating ability with increasing dose in males irradiated as pupae, but not as adults.

## Conclusion

In the past, SIT focused on the induction of almost 100% sterility and this led to the use of high radiation doses. Over the years, it was observed that some insect species irradiated with these high doses were not able to suppress the natural population due to a lack of competitiveness. A revision of requiring 100% sterility was needed and it is suggested that more sterility can be induced in the target population if insects are more competitive when subjected to semi-sterilising doses [2,35]. Male competitiveness at these lower doses needs to be estimated in a semi-field setting, where irradiated males compete with non-irradiated wild males for wild females. On the basis of such experiments, the optimal dose for the released male mosquitoes can be identified. On the other hand, reduced competitiveness can be compensated for by increasing the flooding ratio of sterile males in the field [36].

The choice of developmental stage for irradiation in an SIT programme depends on numerous factors including handling, survival, sterility, competitiveness and release methodology and this study has focused on the first three factors. The mosquitoes in this study were irradiated in small numbers and both stages survived the handling and irradiation process well. Up-scaling the irradiation process for mass production remains a challenge. Pupal irradiation has a number of advantages over adults but not enough resources have been directed to the development of large-scale irradiation devices to draw conclusions.

The longevity of irradiated males was similar to the controls in the adult stage. In the pupal stage, overall similar or higher survival was observed compared to the control. The possibility that irradiation has a beneficial effect on longevity in the pupal stage cannot be excluded from these results, yet longevity was not measured under stressful conditions. In the Mediterranean fruit fly *Ceratitis capitata *(Wiedemann), it is known that under stress the possible negative effects of radiation tend to become more pronounced. Quality control testing in Mediterranean fruit fly SIT programmes measures longevity after the deprivation of food for some time [37]. Although mosquitoes are more sensitive to complete food deprivation, similar tests can be devised for mosquitoes to assess the impact of irradiation.

In this study, fertility was slightly more sensitive to irradiation exposure in the adult stage than in the pupal stage, but differences were small. The trend to reduced insemination rates at higher doses in the pupal stage suggests that pupae are more somatically damaged by the irradiation process than adults, a finding supported by studies that observed that pupae irradiation reduces competitiveness more so than adult irradiation [22,23]. Competitiveness of pupae irradiated at semi-sterilising doses has hardly been studied; but in *An. stephensi *it was observed that males irradiated as pupae with 80 Gy were 1.7 times more competitive than males irradiated with 120 Gy [7]. Future studies will focus on competition experiments that will include the use of sterilizing and semi-sterilizing doses for both developmental stages.

## Authors' contributions

MEHH planned and conducted the experiments, analysed the data and wrote the first draft of the paper. AP provided expertise on insect irradiation and contributed to the final draft of the paper, and BGJK supervised the work and contributed significantly to the final draft of the paper.
